# CDC Grand Rounds: Improving the Lives of Persons with Sickle Cell Disease

**DOI:** 10.15585/mmwr.mm6646a2

**Published:** 2017-11-24

**Authors:** Mary Hulihan, Kathryn L. Hassell, Jean L. Raphael, Kim Smith-Whitley, Phoebe Thorpe

**Affiliations:** ^1^Division of Blood Disorders, National Center on Birth Defects and Developmental Disabilities, CDC; ^2^Division of Hematology, University of Colorado, Aurora, Colorado; ^3^Department of Pediatrics, Baylor College of Medicine, Houston, Texas; ^4^Comprehensive Sickle Cell Center, The Children’s Hospital of Philadelphia, Pennsylvania; ^5^Office of the Associate Director for Science, CDC.

Approximately 100,000 Americans have sickle cell disease (SCD), a group of recessively inherited red blood cell disorders characterized by abnormal hemoglobin, called hemoglobin S or sickle hemoglobin, in the red blood cells. Persons with hemoglobin SS or hemoglobin Sß^0^ thalassemia, also known as sickle cell anemia (SCA), have the most severe form of SCD. Hemoglobin SC disease and hemoglobin Sß^+^ thalassemia are other common forms of SCD. Red blood cells that contain sickle hemoglobin are inflexible and can stick to vessel walls, causing a blockage that slows or stops blood flow. When this happens, oxygen cannot reach nearby tissues, leading to attacks of sudden, severe pain, called pain crises, which are the clinical hallmark of SCD. The red cell sickling and poor oxygen delivery can also cause damage to the brain, spleen, eyes, lungs, liver, and multiple other organs and organ systems. These chronic complications can lead to increased morbidity, early mortality, or both. Tremendous strides in treating and preventing the complications of SCD have extended life expectancy. Now, nearly 95% of persons born with SCD in the United States reach age 18 years (*1*); however, adults with the most severe forms of SCD have a life span that is 20–30 years shorter than that of persons without SCD (*2*).

## Pediatric Advances in Care and Continued Challenges

Most of the morbidity and mortality among pediatric patients with SCD is associated with pneumococcal sepsis, strokes, and pain crises. In 1986, researchers concluded that oral penicillin prophylaxis should start at age 4 months for children with SCA, because of the high rates of morbidity and mortality associated with sepsis in early childhood, and that screening for SCD should take place in the neonatal period (*3*). As a result, since 2006, newborns are universally screened for SCD in all U.S. states, the District of Columbia, Puerto Rico, and the U.S. Virgin Islands (*4*). Current recommendations for pneumococcal infection prevention also include a series of pneumococcal vaccines (*5*). Infarctive strokes occur in 11% of children with SCA (*6*). In 1992, transcranial Doppler ultrasonography was found to predict which children with SCD had the highest risk for developing a stroke (*7*). A few years later, a study demonstrated that chronic blood transfusions lowered the risk for first stroke in children with an abnormal transcranial Doppler (TCD) result by 92% (*8*). The current clinical recommendations are annual screening with TCD for children ages 2–16 years with SCA and, in an effort to prevent stroke, referral of children with abnormal TCD results to a chronic transfusion specialist (*5*).

Increased access to and utilization of health care services by children with SCD is a key component in decreasing morbidity and mortality. However, recent data from the Maryland Medicaid program found that 38% of children with SCD had not seen a hematologist by age 2 years, and 54% of children aged 12–17 years had not seen a hematologist in 2 years, suggesting that “the ambulatory care of many Medicaid-insured children with SCD might be inadequate” (*9*). Furthermore, although it is known that bone marrow transplantation is a promising cure for SCD, with 93% survival and 91% event-free survival after 5 years of follow-up, only 1,000 patients with SCD worldwide have received an HLA-identical sibling transplant (*10*). These findings indicate that, although gains have been made in the treatment of children with SCD, room for improvement remains.

## The Transfer from Pediatric to Adult Care and Continued Challenges

As persons living with SCD age, issues concerning adherence, treatment, complications, and the health care system become different from those encountered during childhood. With so many variables in play, difficulty often occurs in determining the correlation between these factors and the changes in health status that can take place during and after the transfer from pediatric to adult care. Adolescence, in particular, represents a period of medical vulnerability for persons with SCD, given competing demands of normalcy with peers, increasing autonomy in self-management, and advancing disease. For example, Medicaid data suggest that the period of transition from pediatric to adult care is associated with a rise in complications, including pain crises, pulmonary complications, and use of emergency departments (*9*,*11*). The causes of these increased complication rates are multifaceted and include lack of access to qualified health care providers with an understanding and interest in SCD, changes in insurance coverage, psychosocial factors, and others.

Hydroxyurea is a chemotherapeutic agent that increases the production of fetal hemoglobin and decreases SCD-related complications. In adults with SCA, the annual rate of painful crises was significantly less frequent, and the median times to both first and second crises were longer in patients receiving hydroxyurea than in those receiving placebo (*12*). Hydroxyurea use was also found to lower the occurrence of acute chest syndrome (a vaso-occulsive crisis of the pulmonary vasculature) and the need for transfusion therapy. Hydroxyurea is currently labeled for use in adults but is also prescribed to children with SCA. Although hydroxyurea might reduce the occurrence of SCD-related issues, the burden of chronic organ damage remains increasingly important. Contemporary data indicate chronic organ damage is now the leading cause of death for adults with SCD (*13*).

Most adults with SCD have health care insurance, usually Medicaid or Medicare, or both. Still, gaps in coverage might preclude their accessing care. For example, insurance plans might not cover necessary services or high deductibles might preclude use of services. Intermittent or sporadic coverage can occur because of loss of a job that provided insurance or gain of a job that provides a level of income resulting in ineligibility for income-based programs. This lack of access to expert providers and care can further complicate a patient’s disease course. As with the pediatric SCD population, broad opportunity exists for a multistrategy approach to improve health outcomes for adults with SCD.

## A Health Policy Approach

Increasingly, health policy makers advocate the Triple Aim as a model for improving population health (*14*). The first aim is to improve population health, the second is to enhance patient experience, and the third is to reduce health care costs through eliminating preventable acute care utilization and readmissions. With the Triple Aim as the goal, researchers and policy makers are now trying to determine a way to achieve these aims for the SCD community that aligns with current health care priorities and occurs at the individual, provider, and health care system levels. Insufficient data, however, have limited recent efforts to incorporate SCD into health policy initiatives. For example, Healthy People 2020 contained 10 new objectives focused on sickle cell disease (BDBS-1–10); however, all were “archived due to lack of a viable data source” (*15*).

## A Community Approach

The only national community-based organization for SCD, the Sickle Cell Disease Association of America (SCDAA), focuses on improving the quality of life of persons with SCD and finding a cure. SCDAA initiatives include advocacy for increased access to high-quality health care across the lifespan, increased drug development and therapeutic interventions to decrease disease-related complications, and increased availability of low-risk cures for all persons with SCD. To accomplish these goals, SCDAA developed Get Connected, an information-sharing, patient-powered registry. Through this web-based platform, multiple stakeholders can receive information important to the sickle cell community, such as new therapies, opportunities for enrollment in clinical trials, research results, and the locations of knowledgeable providers. The database and network include children and adults with SCD, families, community members, community-based organizations, health care providers, and government and private industry stakeholders.

## A Public Health Approach

The shortage of long-term follow-up programs, registries, or data collection systems has limited the understanding of SCD. To address this gap in knowledge, in 2015, CDC implemented the Sickle Cell Data Collection program to address the need for this public health approach of improving health outcomes. Using state-based surveillance systems, the program provides important population-level data about disease course and the impact of interventions, health care use, and premature death and identifies providers and sites of care. Understanding the onset and progression of complications helps when planning strategies for prevention, early detection, and intervention. The four main objectives of the Sickle Cell Data Collection program are to 1) establish a health profile of the SCD population in the United States; 2) track changes in the SCD population’s outcomes over time; 3) ensure that the SCD community has credible, scientifically sound information to inform standards of care; and 4) inform policy and health care changes. By achieving these goals, the program could improve quality of life, life expectancy, and health of persons living with SCD.

Without data and mechanisms to track and understand SCD care and outcomes, evidence of what works and where improvements could be made is limited. These two current efforts, Get Connected and the Sickle Cell Data Collection program, along with adequate resources and support, have the potential to provide the evidence base to inform health care policies and improve the lives of persons living with SCD.
